# Transactions of American Medical Congress

**Published:** 1878-02

**Authors:** 


### Books Reviewed.

*Transactions of the International Medical Congress of Philadelphia*, 1876. Edited for the Congress by JOHN ASHHURST, JR., A. M., M D., etc. Philadelphia: 1877. Price $6.00.

The first glance which one gives to this volume does not leave a very favorable impression of it as a sample of American book making, but the preface explains that the publication committee preferred to confine themselves within the limits of the amount of money at their disposal, rather than make another assessment as they were empowered to do by vote of the Congress.

The Medical Congress of 1876 was such an important assemblage, that a record of its deliberations will be an addition to medical literature of no small interest or value. The volume opens with a history of the Centennial Medical Commission and the International Medical Congress, prepared by Dr. James H. Hutchinson, of Philadelphia, from which it appears that the project of holding the Congress was first broached in October, 1872, at a meeting of the Philadelphia County Medical Society. In March, 1875, the Centennial Medical Commission was formed with Prof. Gross as President. This commission, together with delegations from the various medical organizations in Philadelphia, had the arrangements for the Congress to perfect, a task which was by no means an easy one to accomplish. The transactions proper of the Congress open with an address of welcome by Prof. S. D. Gross, M. D., LL. D., D. C. L. Oxon., as President of the Centennial Medical Commission. The address was a dignified, graceful and hearty welcome to the members of the Congress, both Foreign and American, which in its full force can only be appreciated by those who heard the distinguished surgeon's remarks. Following the address of welcome, are the minutes of the

Congress, which give a resume of each days proceedings, with the conclusions reached by the various Sections and reported to the Congress. Next after the minutes follows the addresses which were delivered before the Congress in general session. The first of these is the address on Medicine and Medical Progress in the United States, by Prof. Austin Flint, M. D., of New York, is a review of the work of a century in the progress of medical science, in which the author has presented with all of his accustomed force and elegance of diction the salient points in the medical history of the United States. With characteristic modesty he fails to mention any of the numerous contributions to the science which have emanated from his own pen and brain; for instance, he dismisses his discovery of the communicability of typhoid fever by contaminated drinking water, in a sentence of three lines indicating the locality of the discovery, but giving no hint of the discoverer. The address throughout is an impartial, and as far as possible, comprehensive consideration of the subject. Following the address of Dr. Flint is that of Dr. Henry I. Bowditch, of Massachusetts, upon Hygiene and Preventive Medicine. Dr. Bowditch follows the lead of the other speakers, and pays special attention to what has been done in the line of State Medicine during the first century of the republic. He divides the period into three epochs, viz.: I. From 1776 to 1832, the era of theory and dogmatism. II. From 1832 to 1869, that of strict observation, and of bold, often reckless skepticism. III. From 1869 to 1876, that in which the idea of preventive medicine is fairly established. Dr. Bowditch discusses intelligently the condition of Public Hygiene at the present time, and evinces a thorough acquaintance with the subject. By means of an extended and laborious correspondence he presents a report of the status of Preventive Medicine in the various states and territories, and draws from the knowledge thus obtained some very important conclusions. The entire address is one which should be read by all medical men, and which the intelligent portion of the community outside of professional ranks will find well worth a careful perusal. We are glad to see that it has been reprinted and, together with an extensive appendix, issued in book form.

The address of Prof. Theo. G. Wormley, M. D., etc., on Medical Chemistry and Toxicology, is of absorbing interest throughout. His review of the advances which have been made with giant strides by chemical science, and the impetus which the discoveries Black, Priestley, Scheele and Lavoisier, at the opening of the century, gave to careful study of chemistry, is of much interest at this period. The address, after reviewing the advances in medical chemistry and toxicology, concludes with some practical and timely remarks upon the importance of the almost accuracy and honesty on the part of the toxicological chemist.

The address on Surgery by Prof. Paul F. Eve, M. D., is such as would be expected from one who throughout a long and active life kept himself thoroughly posted on American Surgery. While there runs throughout this, a is the case with nearly all of the addresses, a vein of what may be called "American brag," the occasion and the subject are ample excuse for all glorification. The address is a fitting farewell to active literary labor in the field of medicine, and is, we believe, the last important writing by Dr. Eve, whose life has so lately closed, full of years and honors.

Dr. Tyner's address on Medical Biography is as complete, as far as we can judge, as could be desired, and shows the thorough acquaintance of its learned author with American Medical History. Following the address on biography is that on Obstetrics by Prof. Theophilus Parvin, M. D., of Indiana. Dr. Parvin's address, as is the case with anything that emanates from his pen, is forcible, terse and to the point. He deals with the subject as completely as could be desired, in fact, goes far beyond the bounds of Obstetrics and enters upon the field of gyæcological surgery, in which American operators stand prominent. A brief portion of the address is also devoted to Diseases of Children. One of the most interesting addresses, because upon a subject too much neglected by the profession, is that upon Medical Jurisprudence, by Prof. Sanford E. Chaillé, A. M., M. D., of Louisiana. Prof. Chaillé's address deals with the subject from what may be termed the dark ages of medicine, when witchcraft was believed in, the power of demons recognized, and superstition led the judges, priests and physicians to commit deeds upon which we now look with abhorrence and wonder. The author urges upon the profession, with honest and needed zeal, the importance of the addition of medical jurisprudence to the cirriculum of our medical schools. He reviews the rise and progress of the science in the United States, and refers to the more important works published in America upon the topic. The Medico-Legal Society of New York is alluded to in terms of well deserved commendation. A complete bibliography which is appended to the address will be of untold value to students of medical jurisprudence.

The address of Dr. John P. Gray, of Utica, N. Y., upon Mental Hygiene, is a production which as a literary effort deserves high rank, and as a scientific paper is of great value. We wish that the paper could be placed in the hands of every thoughtful man and woman in the country. The tribute which he pays to the mental and moral stamina of the Puritan fathers of New England is, to our mind, one of the finest points of the address. The subject of mental hygiene is here treated by a master hand, by one who has made himself master of the subject, as far as years of study and observation would allow, and his warning notes here sounded in no uncertain tone we cannot as a people afford to neglect.

We have only space to call attention to the addresses on Medical Literature, by Dr. L. P. Yandell, of Kentucky, and on Medical Education, by Dr. N. S. Davis, of Chicago. Dr. J. J. Woodward, Surgeon U. S. A., in an address delivered during an evening session, gave a demonstration of the work of the Medical Staff of the United States Army, which will have a tendency to increase the high reputation already gained by the medical officers of the army, both at home and abroad.

We have not space at this time to consider the work of the sections in detail, and shall, therefore, defer further notice until our next issue. B.

